# Prophylactic antibiotic regimens in tumor surgery (PARITY) survey

**DOI:** 10.1186/1471-2474-13-91

**Published:** 2012-06-07

**Authors:** Khaled Hasan, Antonella Racano, Benjamin Deheshi, Forough Farrokhyar, Jay Wunder, Peter Ferguson, Ginger Holt, Herbert Schwartz, Brad Petrisor, Mohit Bhandari, Michelle Ghert

**Affiliations:** 1 Department of Surgery, McMaster University, Hamilton, ON, Canada; 2 Department of Surgery Surgery, University of Toronto, Toronto, ON, Canada; 3 Department of Orthopedic Surgery, Vanderbilt University, Nashville, TN, USA; 4 Department of Clinical Epidemiology and Biostatistics, McMaster University, Hamilton, ON, Canada

## Abstract

**Background:**

Deep infection following endoprosthetic limb reconstruction for sarcoma of the long bones is a devastating complication occurring in 15% of sarcoma patients. Optimizing infection protocols and conducting definitive surgical trials are critical to improving outcomes. In this study, the PARITY (**P**rophylactic **A**ntibiotic **R**egimens **i**n **T**umor Surger**y**) investigators aimed to examine surgeon preferences in antibiotic prophylaxis and perceptions about current evidence, as well as to ascertain interest in resolving uncertainty in the evidence with clinical trials.

**Methods:**

We used a cross-sectional survey to examine current practice in the prescription of prophylactic antibiotics in Musculoskeletal Tumor Surgery. The survey was approved by our institution’s Ethics Board and emailed to all Active Members of the Musculoskeletal Tumor Society (MSTS) and Canadian Orthopaedic Oncology Society (CANOOS). Survey answers were collected using an anonymous online survey tool.

**Results:**

Of the 96 surgeons who received the questionnaire, 72 responded (75% response rate (% CI: 65.5, 82.5%)). While almost all respondents agreed antibiotic regimens were important in reducing the risk of infection, respondents varied considerably in their choices of antibiotic regimens and dosages. Although 73% (95% CI: 61, 82%) of respondents prescribe a first generation cephalosporin, 25% favor additional coverage with an aminoglycoside and/or Vancomycin. Of those who prescribe a cephalosporin, 33% prescribe a dosage of one gram for all patients and the reminder prescribe up to 2 grams based on body weight. One in three surgeons (95% CI: 25, 48%) believes antibiotics could be discontinued after 24 hours but 40% (95% CI: 30, 53%) continue antibiotics until the suction drain is removed. Given the ongoing uncertainty in evidence to guide best practices, 90% (95% CI: 81, 95%) of respondents agreed that they would change their practice if a large randomized controlled trial showed clear benefit of an antibiotic drug regimen different from what they are currently using. Further support for a clinical trial was observed by an overwhelming surgeon interest (87%; 95% CI: 77, 93%) in participating in a multi-center randomized controlled study.

**Conclusion:**

The current lack of guidelines for the prescription of prophylactic antibiotics in Musculoskeletal Tumor Surgery has left Orthopaedic Oncologists with varying opinions and practices. The lack of current evidence and strong surgeon support for participating in a definitive study provides strong rationale for clinical trials.

## Background

Limb salvage surgery is the standard of care for the vast majority of patients with long-bone sarcoma such as Ewing’s Sarcoma and Osteosarcoma [1]. Limb-salvage reconstruction techniques generally involve replacement of the affected bone segment and joint with a prosthetic implant designed to recreate the normal anatomy. Preservation of neurovascular structures and the need to avoid tumor contamination of the wound result in lengthy, complicated procedures. In addition, many patients require pre- and post-operative chemotherapy for systemic disease management. These factors result in a high incidence of deep post-operative infections (15%–30%) requiring surgical intervention and eventual limb-salvage failure (amputation) in approximately 50% of cases [2-6].

The high infection rates have been stable over the past 2 decades [2,3,5,7]. The standard guidelines for antibiotic prophylaxis as dictated by the American Academy of Orthopaedic Surgeons (AAOS) for total joint replacements includes a pre-operative dose of antibiotics with gram-positive coverage followed by 24 hours of post-operative antibiotics [7,8]. In fact, the only dose that has been shown to be important in preventing post-operative infections is the pre-operative dose [7,9,10].

In higher-risk cases of tumor prosthesis surgery, no guidelines exist to direct antibiotic management. In fact, many tumor surgeons prescribe several days of antibiotics and add gram-negative coverage. However, there is no data to support these practices and the issue of antibiotic resistance with over-prescription therefore becomes important. There has been a clear link between antibiotic overprescription and the emergence of resistant microbial organisms [8,9,11].

To explore current practice in the regimens of antibiotic prophylaxis used for tumor surgery, we conducted an international survey of practicing orthopaedic oncology surgeons in order to learn about their preferences with regards to this practice and to identify the need for future research in this area. Furthermore, we reasoned that the results of this survey may identify factors that influence a surgeon's preference for a particular regimen, serve to educate the orthopaedic community on issues regarding prosthesis infection, and allow for the development of future clinically related trials, which could help develop an international guideline for prophylactic antibiotic regimens in musculoskeletal tumor surgery.

## Methods

### Question development

#### Item generation

We developed a questionnaire using focus groups, key informants, and the previous literature.

The items generated from the focus group were improved by data from a MEDLINE search of articles published from 1975 to 2011 using text words "infection," "sarcoma," "surgery," and “antibacterial agents”. Further items were generated with key informants. Surgeons specializing in orthopaedic oncology provided additional input into potential items for the questionnaire.

#### Pretesting and validity assessments

In order to accurately address the need for antibiotic prophylaxis guidelines in tumour prosthesis surgery, the questionnaire was pretested amongst an independent group of four orthopaedic oncologists (face validity) with respect to reconstruction type, antibiotics used, and time points and dosages administered (content validity). These surgeons also commented on the clarity and comprehensiveness of the questionnaire.

The questionnaire itself consisted four sections, and presented closed-ended questions as multiple-choice or five-point Likert scale formats. Section A encompasses eight questions relating to surgeons’ backgrounds (i.e. age, gender, years in practice, type of practice [academic versus community setting], fellowship training in orthopaedic oncology, and supervision of resident trainees) and surgical volume. Section B sought information regarding surgeons’ management of oncology cases - specifically with regards to how long after chemotherapy can surgery be safely performed, at what white blood cell count the patient is safe for surgery. Section C consists of nine questions that address features of a particular surgeon’s antibiotic regimen (i.e. reconstruction type, specific antibiotics used, time period [pre- and/or post-operatively] and dosages administered). Section D addresses the need for future research in this area and provides participants with an opportunity to offer comments and/or suggestions.

#### Questionnaire administration

All Active Members of the Musculoskeletal Tumor Society (MSTS) and all members of the Canadian Orthopaedic Oncology Society (CANOOS) (duplicates were excluded) were surveyed voluntarily via a web-based method (Survey Monkey). Potential participants were sent the survey by a party independent of the study’s investigators, with no monetary incentives. One e-mail pre-notification was provided. All response data was collected anonymously and grouped according to pre-defined analyses. The Ethics Review Board in conjunction with Hamilton Health Sciences approved this study. No monetary incentives or pre-notification telephone calls were used for this survey. Individual responses were kept confidential and questionnaire completion was voluntary.

#### Sample size

We had a response rate goal of 70% to ensure the results would be adequately powered to prevent the biased ascertainment of outcomes due to non-responder bias [12-14]. To determine the number of respondents needed to sufficiently power our analysis, we assumed that approximately 40% of surgeons surveyed used prophylactic antibiotics until the suction drain is removed for long-bone reconstruction postoperatively. Using the following formula:

(1) $$\begin{array}{l} N=(z^{2}*(p\left(1-p\right)/w^{2}\\ N=\left(1.96^{2}*0.4*0.6\right)/0.05^{2}\\ N=368.8 \end{array}$$

Where:

**N** = required sample size

**Z** = z value (1.96 for 95% confidence interval)

**w** = the confidence interval, expressed as

decimal (0.05 = +/− 5)

**p** = percentage picking a choice (until suction drain removed), expressed in decimal (40% = 0.40)

It was calculated that 369 completed questionnaires would be required to produce a 95% confidence interval (CI) of +/− 5% around the percentages of postoperative prescription of prophylactic antibiotics until suction drain is removed, with an alpha level of 0.05. A total of 96 surgeons were approached to participate. The response rate was 75% (95% CI: 66%, 84%). The sample size of 72 completed survey allowed a 95% CI of +/− 12% around the percentages of the use prophylactic antibiotic until suction drain is removed for long-bone reconstruction postoperatively.

#### Statistical analysis

A previous report has shown that closed-ended questions result in fewer incomplete questionnaires than open-ended formats [15]. The current questionnaire framed the response options in one of two ways: five-point Likert scales or nominal scales. The proportion of participants for each multiple-choice answer with 95% confidence intervals (CI) using Wilson’s exact method were calculated.

## Results

### Characteristics of the respondents

Of the 96 surgeons who received the questionnaire, 72 (75%; 95% CI: 65.5, 82.5%) responded. The typical respondent was a surgeon over 40 yrs old with over 5 yrs in practice (Table 1). The majority (86.1%) work in an academic centre and supervise trainees (90%)] (Table 1). Ninety-seven percent of respondents had completed further orthopaedic oncology fellowship training and 70% of respondents spend greater than 50% of their practice treating orthopaedic oncology patients (Table 1).

**Table 1:** Physician demographics

**Characteristic**		**No.**	**(%)**
Age	Less than 30	0	(0%)
	30–40	12	(16.7%)
	41–50	25	(34.7%)
	51–60	27	(37.5%)
	Over 60	8	(11.1%)
Number of years in practice	Less than 5	3	(4.2%)
	05–10	20	(28.2%)
	11–15	9	(12.7%)
	16–20	13	(18.3%)
	Over 20	26	(36.6%)
Type of Hospital	Academic	62	(86.1%)
	Non-Academic	10	(13.9%)
Supervise residents in training	Yes	65	(90.3%)
	No	7	(9.7%)
Completed a fellowship in Orthopaedic Oncology	Yes	69	(97.2%)
	No	2	(2.8%)
Proportion of practice with bone or soft-tissue tumors	0–25%	5	(5.6%)
	26–50%	17	(23.6%)
	51–75%	12	(16.7%)
	76–100%	39	(54.2%)
Number of long bone sarcomas treated per year	0	0	(0%)
	1–5	8	(11.4%)
	6–10	12	(17.1%)
	11–15	12	(17.1%)
	16–20	16	(22.9%)
	21–25	9	(12.9%)
	> 25	13	(18.6%)

#### Management preferences

Over 90 % (95% CI: 90, 99%) of respondents believe that prophylactic antibiotic protocols are important to decrease the risk of infection in all long bone sarcoma reconstruction types. Forty-six percent (95% CI: 35, 57%) believe that preoperative antibiotic administration is the single most important initial step to preventing postoperative infection while 50% (95% CI: 38, 61%) believe that both preoperative and post-operative antibiotic administration are important to prevent postoperative infection.

#### Antibiotic regimens

Seventy-three percent (95% CI: 61, 82%) of respondents routinely prescribe gram-positive coverage alone for long bone reconstruction while 11% prescribe gram positive and gram negative coverage. Four percent (95% CI: 1, 12%) of respondents prescribe Vancomycin alone. When comparing regimens to gram positive coverage alone, 50% (95% CI: 38, 62%) responded it would be equivalent to a combination of gram positive and gram negative prophylaxis (Table 2).

**Table 2:** Effectiveness of antimicrobial coverage relative to gram positive coverage alone in reducing infection risk in long-bone reconstruction

**Antimicrobial Coverage**	**Definitely less** effective than Gram + alone	**Moderately less** effective than Gram + alone	**Equivalent** to Gram + alone	**Moderately more** effective than Gram + alone	**Definitely more** effective than Gram + alone
**Gram + and Gram - coverage**	2 (3.0%)	2 (3.0%)	33 (50.0%)	23 (34.8%)	6 (9.1%)
**Gram - coverage alone**	40 (60.6%)	16 (24.2%)	10 (15.2%)	0 (0.0%)	0 (0.0%)
**Vancomycin**	3 (4.6%)	6 (9.2%)	31 (47.7%)	22 (33.8%)	3 (4.6%)

#### Duration of antibiotics

Thirty-six percent (95% CI: 25, 48%) of respondents practice discontinuing antibiotics after 24 hrs and 18% (95% CI:11, 29%) discontinue at 48 hours. However 41% (95% CI: 30, 53%) continue antibiotics until the suction drain is removed (Table 3). Forty-three percent (95% CI: 32, 55%) of respondents believe that there is currently no evidence to guide surgeons regarding the optimal antibiotic prophylaxis in long-bone reconstruction.

**Table 3:** Length of time prophylactic antibiotics prescribed following long-bone reconstruction

**Type of Reconstruction**	**24 hours**	**48 hours**	**3–7 days**	**Until suction drain is removed**
**Tumor prosthesis**	25 (35.7%)	13 (18.6%)	3 (4.3%)	29 (41.4%)
**Allograft**	18 (26.5%)	13 (19.1%)	11 (16.2%)	26 (38.2%)
**Allograft-prosthesis composite**	17 (25.4%)	12 (17.9%)	9 (13.4%)	29 (43.3%)

#### First generation cephalosporin dosing

Thirteen percent (95% CI: 7, 23%) of surgeons responded to prescribing one gram of Ancef (Cefazolin) per dose, while 33% (95% CI: 23, 44%) prescribe two grams of Ancef per dose (Table 4). Another 53% (95% CI: 41, 64%) responded that they prescribe two grams of Ancef per dose if the patient weighed greater than 80 kg (Table 4).

**Table 4:** Dosage of Ancef (first generation cephalosporin) prescribed (if applicable)

**Dosage**	**No. (%)**
**1 g**	9 (12.9%)
**2 g**	23 (32.9%)
**2 g only if patient >80 kg**	37 (52.9%)
**N/A**	1 (1.4%)

#### Need for further research

There was a considerable amount of support among respondents for further research including strong support for a large clinical trial to evaluate outcomes following different prophylactic antibiotic regimens. Specifically 84% (95% CI: 73, 91%) felt there was a need for further trials to evaluate outcomes following different prophylactic antibiotic drugs (Table 5). Eighty-three percent (95% CI: 73, 91%) felt there is a need for further trials to evaluate outcomes following different prophylactic antibiotic regimens (Table 5). An overwhelming 90% (95% CI: 81, 95%) of respondents would change their practice if a large randomized controlled trial showed clear benefit of an antibiotic drug and regimen different from what they currently prescribe (Table 6) and 87% (95% CI: 77, 93%) of respondents agreed they would participate in a large randomized control trial. The majority of respondents feel that as little as a 10% absolute risk reduction in infection would be a clinically significant benefit (Table 6).

**Table 5:** Need for further research

	**Strongly agree**	**Agree**	**Unsure**	**Disagree**	**Strongly disagree**
I feel there is a need for further trials to evaluate outcomes following different prophylactic antibiotic drugs	30 (43.5%)	28 (40.6%)	5 (7.2%)	3 (4.3%)	3 (4.3%)
I feel there is a need for further trials to evaluate outcomes following different prophylactic antibiotic regimens	32 (46.4%)	26 (37.7%)	5 (7.2%)	4 (5.8%)	2 (2.9%)
I feel there is a need for studies on the cost-effectiveness of different antibiotic drugs and regimens	18 (26.1%)	28 (40.6%)	11 (15.9%)	5 (7.2%)	7 (10.1%)
I would change my practice if a large randomized controlled trial showed clear benefit of an antibiotic drug and regimen different from what I am currently using	51 (72.9%)	12 (17.1%)	3 (4.3%)	1 (1.4%)	3 (4.3%)

**Table 6:** Clinical importance and interest in participating study

		**No. (%)**	
	Any reduction at all	15	(21.7%)
Amount an alternative antibiotic drug and regimen needs to reduce infection rate before the improvement is considered "clinically important"	5%	10	(14.5%)
	10%	15	(21.7%)
	15%	4	(5.8%)
	20%	12	(17.4%)
	25%	8	(11.6%)
	30%	1	(1.4%)
	35%	0	(0.0%)
	40%	0	(0.0%)
	50%	2	(2.9%)
	>50%	2	(2.9%)
I would participate in a multi-centre randomized controlled study assessing different antibiotic regimens in long-bone reconstruction for tumor surgery.	Yes	61	(87.1%)
	No	9	(12.9%)

## Discussion

The results of this survey demonstrated five key findings. (1) Surgeons vary considerably in their choices of antibiotic regimens and dosages demonstrating a lack of consensus on which prophylactic antibiotic regimen is believed to be most effective. (2) The duration of prescribed regimen varies between surgeons as well with one in three surgeons believing antibiotics could be discontinued after 24 hours while 40% continue antibiotics until the suction drain is removed. (3) Surgeons also felt there was a lack of evidence and uncertainty with regard to which prescription of antibiotic regimen was most effective. (4) Respondents were overwhelmingly in favor of a large multi-center randomized control trial to assess the efficacy of different regimens. (5) More significantly ninety percent of surgeons agreed that they would change their practice if a large randomized controlled trial showed clear benefit of an antibiotic drug regimen different from what they are currently using with the majority of Orthopaedic Oncology Surgeons considering a 10% absolute risk reduction in infection rates to be clinically significant.

These results signify a significant lack of evidence and guidelines directing the prescription of prophylactic antibiotic regimens in musculoskeletal tumor surgery. With the emergence of resistant antimicrobial organisms and outbreaks of *clostridium difficile* in healthcare facilities, duration and prescription of antibiotics has proven to be an important clinical entity. At the same time, a high infection rate of 15-30% reported in many studies [2-6] highlights the importance of optimizing antibiotic regimens. There is no doubt that the development of clinical guidelines is of paramount and immediate importance.

Due to the fact that bone sarcomas are rare, a randomized clinical trial designed to create high level evidence would require multi-institutional and likely international participation. To date, this type of study has not been attempted in the Orthopaedic Oncology community. However, such a study is possible with support from a Surgical Trials Methods Center which exists at the institution of the primary authors for this study. A trial has been designed and is under funding and ethics review, which will involve randomizing patients undergoing lower extremity tumor prosthesis reconstruction to either 24 hours or 5 days of cephalosporin coverage. The study will be double-blinded as randomization will be completed by the Pharmacy Department at each institution. Thus, with completion of this study, there is the possibility that eventually guidelines such as those provided by the AAOS will be created for this very challenging peri-operative issue in Orthopaedic Oncology.

The strengths of our study include obtaining a comprehensive sampling of North American orthopaedic oncology surgeons from both the Canadian Orthopaedic Oncology Society (CANOOS) and Musculoskeletal Tumor Society (MSTS), achieving an exceptional survey response rate of approximately 75% that helps to limit non-responder bias with active surgeon participation along with a comprehensive sampling of surgeons from academic and non-academic centres,

Our response rate of 75% of Orthoepdic Oncologists provided a robust data set for the general purposes of our study as well as exceeded the level for our anticipated study precision. Nevertheless, future studies that are aimed at more rigorously evaluating potential sampling- bias will include surgeons from Europe and Asia. While non-responeder bias could not be eliminated it was minimized through re-administering the survey specifically to those who had yet to complete the survey.

## Conclusion

We have shown that there are varied opinions and variations from surgeons on the prescription of prophylactic antibiotic regimens in tumor surgery. There is as yet no clinical trial on the efficacy of different antibiotic regimens in preventing infection in long-bone prosthetic reconstruction. However, there is overwhelming support in the Orthopaedic Oncology community to participate in a multi-center trial, which is currently in the state of development.

## Competing interests

The author(s) declare that they have no competing of interest.

## Authors' contributions

KH carried assisted in the conception and design of the survey as well as data acquisition. KH assisted in analyzing and interpreting the data as well as drafting and revising the final manuscript. AR assisted in developing the figures and participated in revising the manuscript. BD assisted in study design, providing background knowledge as well as revising the manuscript. FF assisted in data analysis and interpretation. JW, PF, BP, and MB provided critical revisions to the manuscript as well as assisting in developing study design. MG conceived and designed the survey and the study. MG provided critical revisions and assisted in drafting the manuscript as well as approving the final manuscript for submission for publication.

## Pre-publication history

The pre-publication history for this paper can be accessed here:

http://www.biomedcentral.com/1471-2474/13/91/prepub
